# Music-evoked incidental happiness modulates probability weighting during risky lottery choices

**DOI:** 10.3389/fpsyg.2013.00981

**Published:** 2014-01-07

**Authors:** Stefan Schulreich, Yana G. Heussen, Holger Gerhardt, Peter N. C. Mohr, Ferdinand C. Binkofski, Stefan Koelsch, Hauke R. Heekeren

**Affiliations:** ^1^Cluster of Excellence “Languages of Emotion,” Freie Universität BerlinBerlin, Germany; ^2^Department of Education and Psychology, Freie Universität BerlinBerlin, Germany; ^3^Department of Neurology, University Hospital of Schleswig-HolsteinLübeck, Germany; ^4^Center for Economics and Neuroscience, University of BonnBonn, Germany; ^5^Department of Psychology, Universität KonstanzKonstanz, Germany; ^6^Division for Clinical Cognitive Sciences, Department of Neurology, RWTH Aachen UniversityAachen, Germany

**Keywords:** decision making, happiness, incidental emotions, music, probability weighting, prospect theory, risk, risk aversion

## Abstract

We often make decisions with uncertain consequences. The outcomes of the choices we make are usually not perfectly predictable but probabilistic, and the probabilities can be known or unknown. Probability judgments, i.e., the assessment of unknown probabilities, can be influenced by evoked emotional states. This suggests that also the weighting of known probabilities in decision making under risk might be influenced by incidental emotions, i.e., emotions unrelated to the judgments and decisions at issue. Probability weighting describes the transformation of probabilities into subjective decision weights for outcomes and is one of the central components of cumulative prospect theory (CPT) that determine risk attitudes. We hypothesized that music-evoked emotions would modulate risk attitudes in the gain domain and in particular probability weighting. Our experiment featured a within-subject design consisting of four conditions in separate sessions. In each condition, the 41 participants listened to a different kind of music—happy, sad, or no music, or sequences of random tones—and performed a repeated pairwise lottery choice task. We found that participants chose the riskier lotteries significantly more often in the “happy” than in the “sad” and “random tones” conditions. Via structural regressions based on CPT, we found that the observed changes in participants' choices can be attributed to changes in the elevation parameter of the probability weighting function: in the “happy” condition, participants showed significantly higher decision weights associated with the larger payoffs than in the “sad” and “random tones” conditions. Moreover, elevation correlated positively with self-reported music-evoked happiness. Thus, our experimental results provide evidence in favor of a causal effect of incidental happiness on risk attitudes that can be explained by changes in probability weighting.

## Introduction

Making decisions under risk is an integral part of our lives: we order meals that we have not tried yet, buy products that we have never used before, and we decide how to invest money for ourselves, for friends, or for customers. In both economics and psychology, risk is often understood as a function of the variability of outcomes. People's attitudes toward this variability differ substantially (see, e.g., Dohmen et al., 2011) and can be characterized by their degree of risk aversion (or risk proclivity, respectively). A risk-averse person prefers a sure outcome over any gamble that has the same expected value; for a risk-loving person, the opposite holds (Wakker, 2010, p. 52). For instance, a risk averter prefers €5 for sure over the gamble that pays €10 with a probability of 75% and −€10 with 25% probability.

In (cumulative) prospect theory (Kahneman and Tversky, 1979; Tversky and Kahneman, 1992), risk attitudes expressed in people's decisions are attributed to several constructs that describe how the available options are subjectively evaluated. The three constructs are (1) comparison of the objective outcomes with a reference point, (2) transformation of the resulting gains and losses into subjective values, and (3) transformation of the objective probabilities associated with the possible outcomes into subjective decision weights for those outcomes. The two subjective transformations are formalized by the value function and the probability weighting function, respectively. Both functions are thought to reflect the often observed psychophysical characteristic of diminishing marginal sensitivity, i.e., less sensitivity to changes in outcomes and probabilities, the farther they are away from the respective reference points. This results in a convex value function for losses and a concave value function for gains. For gains and losses, the reference point can be, for instance, the status quo (i.e., the current wealth level). For probability weighting, the extreme cases of impossibility (*p* = 0) and certainty (*p* = 1) are the two natural points of reference (Fox and Poldrack, 2013). This results in an inverse S-shaped form of the probability weighting function, reflecting the common empirical finding that small probabilities are overweighted and large probabilities are underweighted.

Studies that used semiparametric (Abdellaoui et al., 2011) or parametric (Fehr-Duda et al., 2010) specifications of the value and the probability weighting function suggest that probability weighting is more susceptible to situational influences than outcome valuation. As a consequence, there is increasing interest in the factors that determine the shape of the probability weighting function—especially its two main characteristics, curvature and elevation (see the discussion in Gonzalez and Wu, 1999).

One important factor that influences probability weighting seems to be affect, as several theoretical accounts of the determinants of probability weighting suggest. According to one account, the commonly observed inverse S-shape of the probability weighting function results from anticipated elation or disappointment regarding the future realization of an uncertain payoff (Gul, 1991; Brandstätter et al., 2002; Walther, 2003). For instance, one might anticipate disappointment from a failure to achieve a highly probable gain. This in turn is thought to translate into decision weights for high probabilities that are lower than the actual probabilities.

In a similar vein, Rottenstreich and Hsee (2001) hypothesized that the extent of probability weighting depends on the “affective richness” of potential outcomes. Confirming their hypothesis, the authors found that “affect-rich” outcomes—i.e., outcomes which participants anticipate to elicit strong emotional reactions (such as receiving an electric shock or a kiss)—were associated with more pronounced probability weighting than less “affect-rich” outcomes (such as receiving money). The authors speculated that hope and fear generated by affect-rich outcomes give rise to the shape of the probability weighting function. Although these studies focused on the curvature of the probability weighting function, it has been pointed out that also the elevation parameter might capture an emotional influence (Rottenstreich and Hsee, 2001).

Importantly, not only emotions related to the decision outcomes might be reflected in probability weighting. Even incidental emotions, which are characterized by being unrelated to the judgments and decisions at issue (Loewenstein and Lerner, 2003; Weber and Johnson, 2009), were found to have an influence on probability judgments, i.e., the assessment of unknown probabilities. For instance, happy people made more optimistic probabilistic judgments and sad people more pessimistic judgments (Johnson and Tversky, 1983; Wright and Bower, 1992). It is thus plausible that similar effects are observable in the subjective weighting of known probabilities in decision making under risk. The elevation of the probability weighting function is thus a promising target of affect, with greater elevation representing more optimistic attitudes and reduced elevation more pessimistic attitudes toward risky situations.

While there is a considerable body of evidence on the influence of incidental emotions on decision making under risk, only a few studies linked incidental emotions specifically to the constructs postulated by cumulative prospect theory (CPT). For instance, Isen et al. (1988) found that positive affect made participants value losses more negatively, while it had no significant effect on the valuation of gains. Thus, positive affect made participants more loss-averse. The authors, however, restricted their design to two-outcome lotteries with 50%/50% probabilities and did not investigate the role of probability weighting. In a recent study, Fehr-Duda et al. (2011) provided correlational evidence that they interpreted as an effect of mood on the elevation of the probability weighting function for both gains and losses in women, but not in men. Women that regarded the current day to be more promising than usual made decisions that are consistent with more optimistic probability weighting. A similar link was also suggested in another study that revealed a correlation between seasonal and weather conditions and probability weighting, which the authors also interpreted as mood effects (Kliger and Levy, 2008).

Studies without direct manipulation and measurement of affective states leave open the question whether incidental emotions are indeed the mediator of the effects mentioned above. To answer this question, it is necessary to establish a causal effect of incidental emotions on risk attitudes that is consistent with probability weighting in particular. One way to prove a causal effect is to experimentally manipulate incidental emotions, record participants' self-reported emotions, and investigate the emotion-induced changes in probability weighting.

To this end, we employed a variant of the Random Lottery Pairs procedure (Hey and Orme, 1994) and manipulated emotions within-subject by playing different types of music to our participants. They listened to happy and sad music as well as to sequences of random tones or to no music at all.

To determine whether the emotion manipulation had an effect on participants' decision making, we compared the frequencies with which they chose the riskier lotteries between conditions. Furthermore, we estimated preference parameters via structural regressions based on CPT and tested whether probability weighting changed between conditions.

Based on the studies that established a link between incidental emotions and optimistic or pessimistic probability judgments (Johnson and Tversky, 1983; Wright and Bower, 1992), we hypothesized that probability weighting in decision making under risk would be affected in a similar way. Specifically, we hypothesized that participants in the “happy” condition exhibit increased probabilistic optimism in the sense that they attach higher decision weights to the larger outcomes. In contrast, listening to sad music should lead to more pessimistic probability weighting, i.e., lower decision weights associated with the larger outcomes. We expected this effect to manifest itself also in a relationship between the self-reported emotional state and the extent of probability weighting.

Because an increased elevation of the probability weighting function implies a reduction in risk aversion (see Wakker, 2010, chapter 5), it follows from these hypotheses that participants should choose the riskier lottery more frequently after listening to happy music than after listening to sad music.

Research has repeatedly demonstrated that the intensity of evoked emotions gradually decreases over time (Isen et al., 1972; Isen and Gorgoglione, 1983; Gard and Kring, 2007; Andrade and Ariely, 2009). Thus, we hypothesized that music-evoked emotional effects on risk attitudes would be strongest at the beginning and then diminish. This would corroborate an affective interpretation of the effects on decision making.

## Materials and methods

### Participants

We recruited 46 participants through bulletin-board appeals at Freie Universität Berlin and an e-mail mailing list to which previous and prospective participants had subscribed. Four participants had to be excluded from the analysis because they did not participate in all sessions. One participant was dropped from the analysis because she stated in the post-experimental questionnaire that she had chosen arbitrarily between the lotteries presented. The remaining 41 participants (28 women; 13 men) had a mean age of 27.37 years (*SD* = 7.832 years). All participants gave written informed consent prior to the experiment.

### Procedure

#### Experimental design

In a within-subject design, participants were exposed to auditory stimulation: (1) happy or (2) sad music or (3) sequences of random tones, while (4) no music was played in the fourth condition. Each of the four experimental conditions was tested in a separate session. The order of the conditions was randomized, and all sessions were one week apart. In three of the conditions, up to four participants were present in the lab simultaneously. Each participant sat in front of a computer equipped with headphones and enclosed in cubicles to prevent eye contact with the other participants during the experiment. All tasks were presented on a computer screen (except the post-experimental questionnaires, which were handed out on paper), and all data were recorded using the software Presentation (Neurobehavioral Systems, Inc.). Responses were made via a standard keyboard.

The experimenter handed out instructions and read them aloud. Subsequently, participants answered a quiz on the instructions to make sure that they had understood the lottery choice task. At the beginning of the music conditions, participants shortly listened to the musical pieces for familiarization. In the “no music” condition, participants filled in the demographic questionnaires.

**Figure 1:**
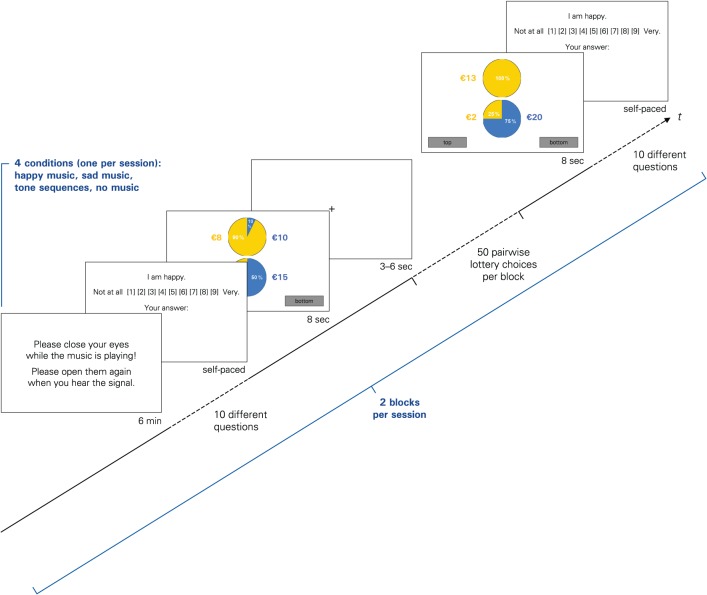
**The sequence of events forming one block in the experiment.** Each condition consisted of two such blocks, separated by a short break.

The main experiment started with the emotion evocation and the emotional rating task (see below). In the “no music” condition, it started with the emotion rating task right away, while in all other conditions, participants first listened to music for exactly 6 min via headphones. The subsequent block of pairwise lottery choices lasted approximately 10 min, comprised 50 trials (plus five initial learning trials), and was followed by the second emotion rating task (see Figure 1). In each trial, participants were asked to choose one of two lotteries within a time frame of 8 s. Participants did not receive any feedback on earnings in-between trials. The trials were separated by a variable interval (3–6 s), which served as a short period of rest and as a means to minimize potential anticipation effects and repetitive behavioral patterns. The entire sequence was repeated—except for the familiarization phase and the learning trials—so that each condition included two music blocks, four emotion ratings tasks (two post-music, two post-choice), and 100 lottery choices in total. The same set of 100 different lottery pairs (see Table A1 in the Appendix) was used in each condition.

At the end of each session, participants filled in a questionnaire concerning their choice strategies and thoughts on the experiment's purpose. None of the participants mentioned any hypothesis concerning an emotion-specific connection between the type of music played and their level of risk aversion. After the final session, participants learned their individual earnings and received them in cash. These consisted of a randomly determined payoff according to the gamble they had chosen in one randomly selected trial plus the total attendance fee of €24 for all four sessions.

#### Musical stimuli

The musical stimuli were chosen to evoke (a) happiness, (b) sadness, and (c) neither happiness nor sadness. The latter stimuli we refer to as “random tones” (for the complete list of stimuli see Table A2). The happy pieces and random tone sequences had been used in a recent study (Koelsch et al., 2013). Half of the sad pieces were used by Pehrs et al. (2013), overlaid with an acoustically identical electronic beat.

The happy pieces consisted of 12 instrumental excerpts of 30 s duration each from various epochs and styles (classical music, Irish jigs, jazz, reggae, South American, and Balkan music). Sad pieces were classical and indie-pop pieces with a duration of 60 s each, selected on the basis of features that have been shown to evoke sad feelings, i.e., minor key, slow tempo, and low pitch variation (Juslin and Laukka, 2004; Lundqvist et al., 2009). The 12 random tone sequences featured acoustically changing stimuli of 30 s duration. These isochronous tones for which the pitch classes were randomly selected from a pentatonic scale (see Koelsch et al., 2013) were created with the help of the MIDI toolbox for MATLAB (Eerola and Toiviainen, 2004).

The manipulation check revealed that the random tones were not affectively neutral (see Results). Consequently, the “no music” condition remains as the one condition in which the affective state was not manipulated.

All stimuli were non-vocal pieces, edited with Praat (version 5.0.29, Boersma, 2002) to feature a 1.5-s fade-in and fade-out and the same intensity (70 dB). The total duration of the sad music pieces matched the total duration of the auditory stimulation in the other conditions (i.e., 6 min).

#### Emotion rating

In the computerized, self-paced emotion rating task, participants reported their current emotional state by indicating the degree to which they agreed with three statements concerning happiness (“I am happy”), sadness (“I am sad”), and calmness (“I am calm”). The latter served as a reverse proxy for arousal. The scale ranged from 1 (“I completely disagree”) to 9 (“I completely agree”). These items correspond to those typically used to infer basic emotions (e.g., in the Differential Emotions Scale, see Izard et al., 1993). Basic emotions have proven to be more informative than the concept of valence alone to study the effect of emotions on risky choices (Lerner and Keltner, 2000, 2001).

To reduce potential experimenter demand effects (Orne, 1962) and to obscure the objective of the emotion ratings from the participants in the sense of “non-deceptive obfuscation” (Zizzo, 2010), seven additional ratings were acquired that were not directly related to basic emotional states (e.g., “I slept well last night”; for the complete list, see Table A3 in the Appendix).

#### Lottery choice task

For the lottery choice task, we used a variant of the Random Lottery Pairs procedure (Hey and Orme, 1994). In each trial *t*, participants were shown a lottery pair {***A***_*t*_, ***B***_*t*_} out of a set of 100 lottery pairs (for the complete list, see Table A1 in the Appendix) in pseudo-random order. The pseudo-random order differed per session/condition and per subject.

Each lottery ***L*** consisted of two possible, strictly positive payoffs (*x*_***L***,1,_ *x*_***L***,2_), denoted in euro, and the associated probabilities (*p*_***L***,1,_ *p*_***L***,2_) = (*p*_***L***,1,_ 1 − *p*_***L***,1_). We limited our study to the gain domain for the following reasons: first, neuroimaging and lesion studies suggest that losses and gains are processed differently in the human brain (Tom et al., 2007; De Martino et al., 2010). Second, to increase the power for the detection of an effect, a sufficient number of decision trials is needed. Third, mixed gambles would have required the estimation of additional parameters, making even more observations necessary. We therefore chose to dedicate all our experimental trials to only one domain.

The payoffs and probabilities were visualized on screen by a pie chart (see Figure 1), which is a common graphical representation of lotteries in this type of experiments (Harrison and Rutström, 2008). Apart from some “catch trials,” we ensured that within each pair, no lottery first-order stochastically dominated the other lottery.

The lotteries differed from each other in their riskiness. A lottery can be considered riskier than another lottery if it can be expressed as a mean-preserving spread (MPS) of the other lottery (Rothschild and Stiglitz, 1970). Since risk averters dislike the wider spread, making them choose the riskier lottery requires adding some compensation for the wider spread—a “risk premium”—to the riskier lottery. We denote this risk premium by *m*. Within a lottery pair {***A***_*t*_, ***B***_*t*_}, we thus call the lottery ***A***_*t*_ the riskier lottery if it has a wider spread than ***B***_*t*_, such that ***A***_*t*_ = MPS(***B***_*t*_) + *m*_*t*_ (*m*_*t*_ being a sure payoff)^1^.

The set of lottery pairs was designed to allow estimating preference parameters with relatively high precision in the range that has been found in previous studies (see, e.g., the examples given in Harrison and Rutström, 2008; Table 5 in Stott, 2006). That is, for degrees of risk aversion usually observed in lab experiments, we expected participants to sometimes choose the riskier and sometimes the less risky lottery. In addition, the payoffs of our lotteries were associated with probabilities spanning 10 to 90% to cover a broad enough range to reliably estimate the parameters of the probability weighting function.

Positioning of the lotteries on screen was counterbalanced within-subject: in some trials, the riskier lottery was presented in the upper half of the screen, and sometimes in the lower one. Moreover, we counterbalanced the position of the larger payoff on screen between-subjects: for half of the participants, the larger payoff was illustrated by the left side, and for the other half, by the right side of the pie chart.

### Statistical analyses

#### Emotion ratings

To check whether the experimental manipulation had the desired emotional effects, we calculated repeated-measures ANOVAs using the four conditions as the within-subject factor. As dependent variables in these ANOVAs we used the ratings in three affective dimensions (happiness, sadness, and calmness). For each dimension, we analyzed the ratings obtained immediately after the musical stimulation (“post-music ratings”). In these analyses, we used the average of the two post-music ratings per condition and per participant. We also calculated an ANOVA for the average post-choice ratings to investigate if emotional effects persisted over time.

#### Lottery choices

***Relative frequency with which the riskier lottery was chosen.*** We analyzed how often the riskier of the lotteries included in a pair was chosen in those trials in which one lottery is riskier than the other according to the measure explained above. This is the most basic measure of the influence of music-evoked emotions on risk attitudes.

These choice frequencies were compared across the four conditions. To establish whether there are significant differences between the four conditions, we estimated linear probability models (LPMs)^2^. That is, we regressed choice of the riskier lottery on condition dummies. Let us denote participant *i*'s choice in trial *t* by *r*_*i,t*_, and set *r*_*i,t*_ = 1 if the riskier lottery was chosen by *i* in trial *t*, and *r*_*i,t*_ = 0 otherwise. The regression equation then is

$$\begin{array}{l} r_{i,t}=\beta_{\text{hap},i}+\delta_{\beta ,\text{nom}}D_{\text{nom},i,t}+\delta_{\beta ,\text{ton}}D_{\text{ton},i,t}\\ \;+\delta_{\beta ,\text{sad}}D_{\text{sad},i,t}+\varepsilon_{i,t}. \end{array}$$

β_hap_ is the relative frequency at which the riskier lottery was chosen in the “happy” condition (which here serves as the reference condition). δ_β,nom_ captures the difference in the choice of the riskier lottery in the “no music” condition vis-à-vis the “happy” condition, while δ_β,ton_ does the same for the “random tone sequences” condition, and δ_β,sad_ for the “sad” condition. *D* is the respective condition dummy regressor, and ε_*i,t*_ is an error term with mean zero. Our regression allowed for heterogeneity in risk aversion in the reference category between subjects *i* via individual random effects in the regression's constant term, here β_hap,*i*_.

A more versatile regression also included regressors *d*_*i,t*_ measuring how many trials had passed since the last musical stimulation. This was done to investigate whether the effect of the evoked emotions on risk attitudes diminished over time. We denote the associated coefficients by τ_β,cond_:

$$\begin{array}{l} r_{i,t}=\beta_{\text{hap},i}+\tau_{\beta ,\text{hap}}d_{i,t}D_{\text{hap},i,t}+\left(\delta_{\beta ,\text{nom}}+\tau_{\beta ,\text{nom}}d_{i,t}\right)D_{\text{nom},i,t}\\ \;+\left(\delta_{\beta ,\text{ton}}+\tau_{\beta ,\text{ton}}d_{i,t}\right)D_{\text{ton},i,t}\\ \;+\left(\delta_{\beta ,\text{sad}}+\tau_{\beta ,\text{sad}}d_{i,t}\right)D_{\text{sad},i,t}+\varepsilon_{i,t}. \end{array}$$

To simplify comparison of the more extensive model with the reduced model, the regressor *d*_*i,t*_ was centered.

Participants failed to respond in only 72 out of 41 × 4 × 100 = 16,400 trials (0.439%), such that we had to omit these trials in the analysis. In the LPMs, the number of observations is lower, since not all trials featured a “risky–less risky” trade-off as defined above via mean-preserving spreads; 70 out of the 100 lottery pairs we used involved a trade-off of this kind (while others involved, e.g., mean–variance trade-offs).

We compared several models, which differed in the number of random effects—i.e., individual random effects were included either only in the baseline risk aversion or also in the between-condition changes and/or in the time trends. The two models described in detail above yielded the lowest Bayesian Information Criteria (BICs).

***Structural regressions.*** To find out whether changes in participants' choices between conditions can indeed be attributed to changes in probability weighting, we estimated structural regression models (see, e.g., Harrison and Rutström, 2008, section 2.2; Wilcox, 2011). These are based on cumulative prospect theory (CPT). In CPT, monetary payoffs and the probability of receiving these payoffs are transformed into subjective values via a value (utility) function *u* and a probability weighting function *w*, respectively.

If participants assign a subjective value *V* to a lottery in line with CPT, probability weighting is applied to the probability of the larger payoff (see Tversky and Kahneman, 1992). That is, if we denote the larger payoff in lottery ***L*** = (*x*_***L***,1,_ *p*_***L***,1_; *x*_***L***,2_, *p*_***L***,2_) by *x*_***L***,1,_ the subjective value *V* is given by

$$V(L;\theta )\equiv w\left(p_{L,1};\theta_{w}\right)u\left(x_{L,1};\theta_{u}\right)+\left[1-w\left(p_{L,1};\theta_{w}\right)\right]u\left(x_{L,2};\theta_{u}\right).$$

**θ** is a vector combining the parameter vectors **θ**_*w*_ and **θ**_*u*_ that determine the shape of the probability weighting function and the shape of the utility function, respectively. It is these parameters and their potential modulation by the emotional state that we are interested in.

Regarding the transformation of the payoffs, we assume—in line with many previous studies (e.g., Tversky and Kahneman, 1992)—a power utility function^3^, i.e.,

$$u\left(x;\theta_{u}\right)=u(x;\rho )=x^{1-\rho },$$

such that a larger ρ goes along with increased curvature of the utility function—i.e., all other things equal, increased risk aversion^4^. For the probability-weighting function, we use a popular two-parameter version (Prelec, 1998),

$$w\left(p;\theta_{w}\right)=w\left(p;\alpha ,\beta \right)=\exp\{-\beta {\left(-\log p\right)}^{\alpha }\}.$$

Here, *w*(*p*; α, β) is the decision weight, *p* is the objective probability, and α and β are parameters. Two-parameter versions of probability weighting have found broad empirical support due to their ability to explain individual differences or differences between choice domains (Gonzalez and Wu, 1999; Abdellaoui, 2000; Bleichrodt and Pinto, 2000; Abdellaoui et al., 2010; Capra et al., 2012). Importantly, the two parameters are moreover thought to reflect different psychological phenomena (see, e.g., Gonzalez and Wu, 1999). The parameter α primarily influences the slope of the probability weighting function: for β = 1, α < 1 results in overweighting of small and underweighting of large probabilities, with the consequence of relative insensitivity [∂ *w*(*p*; α, 1)/∂*p* < 1] in the intermediate range. The parameter β primarily reflects the elevation of the weighting function and can be interpreted as reflecting the “attractiveness” of gambling: for α = 1, β > 1 results in an underweighting of all probabilities [*w*(*p*; 1,β) < p]. That is, in CPT, a less elevated weighting function assigns a lower decision weight to the higher outcome—see the formula for *V*(***L***;ρ, α, β). This has also been interpreted as a form of “pessimism” (in Fehr-Duda et al., 2011). Through the reduced decision weight on the higher lottery outcome, reduced elevation of the probability weighting function translates to greater risk aversion.

Based on this, for each lottery pair {***A***, ***B***}, the difference in the subjective values, Δ *V*(***A***, ***B***; **θ**) ≡ *V*(***A***; **θ**) − *V*(***B***; **θ**), is determined. A decision maker whose preferences can be represented by the subjective value function *V* chooses ***A*** over ***B*** whenever the subjective value of ***A*** is larger than that of ***B***, i.e., Δ *V*(***A***, ***B***; **θ**) > 0, and vice versa. Of course, participants do not make choices that are perfectly consistent with the assumed model. The most frequently used binary-choice regressions—the logit and the probit specification—account for this by mapping the difference in subjective valuation, Δ *V*(***A***, ***B***; **θ**), to choice probabilities via a strictly increasing, symmetric (“sigmoid”) link function *F*. Formally,

$$\begin{array}{l} \text{Pr}[A|\{A,B\};\theta ,\sigma ]=F[\Delta V(A,B;\theta )/\sigma ]\text{ and}\\ \text{Pr}[B|\{A,B\};\theta ,\sigma ]=1-F[\Delta V(A,B;\theta )/\sigma ]. \end{array}$$

We use the logit specification, such that the link function *F* is the logistic distribution function, *F*[Δ *V*] = 1/[1 + *e*^−Δ *V*^]^5^.

The parameter σ governs the dispersion (flatness) of the link function. It is often called the Fechner noise parameter (see Harrison and Rutström, 2008). The larger σ (i.e., the more noise), the smaller the fraction gets, with the effect that σ → ∞ is equivalent to random choice (i.e., *F* → ½). Conversely, σ → 0 means that no noise is present in participants' choices from the perspective of the model, and the choice probabilities converge to a step function.

Based on both theoretical and econometric considerations, it has been suggested to modify this common approach (Wakker, 2010, p. 85; Wilcox, 2011), because it suffers from the fact that the utility assigned to a certain payoff in expected utility theory or CPT is only unique up to an affine transformation (Wilcox, 2011, p. 90). However, the common approach effectively takes the ordinal quantity subjective utility to be a cardinal quantity. Wilcox (2011) shows that this has the consequence that being “more risk-averse” in the theoretical sense (Pratt, 1964) and being “stochastically more risk-averse” do not coincide: it is easy to find pairs, e.g., of a lottery ***B*** and a sure payoff *A* = E[***B***] for which the difference in subjective valuation, Δ V, approaches zero if one increases the degree of risk aversion (ρ ↑). Consequently, the predicted probability of choosing either alternative approaches ½—which is non-sensical, since greater risk aversion (ρ ↑) should imply a predicted probability of choosing the sure payoff that increases and approaches one.

A remedy to this problem is to replace the difference in subjective valuation, Δ V, by the difference between the certainty equivalents of these valuations (Wakker, 2010, p. 85; Von Gaudecker et al., 2011, p. 676)—i.e., sure amounts of money that carry the same subjective value as the lotteries. Under power utility, the certainty equivalent of a subjective value *V* is given by

$$\begin{array}{l} CE(L;\theta )\equiv u^{-1}\left[V(L;\theta );\rho \right].\\ \;=\{\begin{array}{ll} {\left[(1-\rho )V(L;\theta )+1\right]}^{1/(1\;-\;\rho )} & \text{if }\rho \neq 1\\ \exp[V(L;\theta )] & \text{if }\rho =1. \end{array} \end{array}$$

We can then define, for each lottery pair {***A***, ***B***}, the difference in the certainty equivalents, Δ CE(***A***, ***B***; **θ**) = CE (***A***, **θ**) − CE(***B***,**θ**). With this, the specification of the CPT-based latent-variable model becomes:

Pr[A|{A,B};θ,σ]=F[ΔCE(A,B;θ)/σ].

Let ***C***_*t*_ denote the lottery that was actually chosen in trial *t*, and let **1**_***A***_*t*__ be the indicator function such that **1**_***A***_*t*__(***C***_*t*_) = 1 if ***A***_*t*_ was chosen and 0 if ***B***_*t*_ was chosen in *t*. The few trials in which participants failed to respond (72 out of 16,400) are omitted from the analysis. Let *D*_cond,*t*_ be a dummy regressor that equals 1 only in trials *t* belonging to the respective condition cond and 0 otherwise; for instance, when choosing the “happy” condition as the reference condition, the dummy regressors would cover cond ∈{“no music,” “random tones,” “sad music”}. *T* is the total number of trials in the experiment.

Non-linear maximum likelihood estimation maximizes the log-likelihood

$$\begin{array}{l} \ell (\theta ,\Delta_{\theta },\sigma ,\delta_{\sigma })\equiv \\ \;\sum_{t\;=\;1}^{T}\{1_{A_{t}}(C_{t})\log F\left[\frac{\Delta CE\left(A_{t},B_{t};\theta \;+\;\sum_{\text{cond}}\delta_{\theta ,\text{cond}}D_{\text{cond},t}\right)}{\sigma \;+\;\sum_{\text{cond}}\delta_{\sigma ,\text{cond}}D_{\text{cond},t}}\right]+\\ \;\{1-1_{A_{t}}(C_{t})\}\log\left\{1-F\left[\frac{\Delta CE\left(A_{t},B_{t};\theta \;+\;\sum_{\text{cond}}\delta_{\theta ,\text{cond}}D_{\text{cond},t}\right)}{\sigma \;+\;\sum_{\text{cond}}\delta_{\sigma ,\text{cond}}D_{\text{cond},t}}\right]\right\}\}. \end{array}$$

That is, $\left(\hat{\theta },{\hat{\Delta }}_{\theta },\hat{\sigma },{\hat{\delta }}_{\sigma })\equiv \text{arg max}\ell (\theta ,\Delta_{\theta },\sigma ,\delta_{\sigma }\right)$. **θ** and σ are the preference and noise parameters that describe behavior in the reference condition. The matrix **Δ_θ_** and the vector δ_σ_ capture the changes in **θ** and the changes in the Fechner noise parameter σ, respectively, between the reference condition and the three remaining conditions.

We compared a full model that permitted condition-wise changes in both the value function parameter (ρ) and the probability weighting function parameters (α, β) with a more parsimonious model that only allowed for changes in probability weighting. To account for between-subject heterogeneity in the valuation of outcomes and in probability weighting, these regressions allowed for individual random effects in ρ, α, and β. Allowing for changes in the curvature of the value function did not significantly improve the model's fit to the data, as assessed by a likelihood-ratio test. Therefore, we report the parameter estimates of the more parsimonious model in detail. *F*-statistics were calculated, and individual coefficients were tested for significance.

***Complementary structural regressions.*** We investigated the link between incidental emotions and probability weighting in a complementary way by using participants' self-reported happiness ratings as explanatory variables. Specifically, we calculated for each participant the average of the four happiness ratings in the “no music” condition and used this individual average as a between-subject regressor. The average score of the “no music” condition represents baseline happiness, as there was no experimental manipulation of affect in this condition. We then calculated, for each participant, the deviation of the condition-specific happiness ratings (i.e., one value per condition, calculated as the average of the four ratings obtained per condition) from his/her individual baseline happiness; this deviation served as a within-subject regressor.

In other words, this regression allowed us to investigate (a) whether participants who are happier in general exhibit more/less pronounced probability weighting, and (b) whether the music-evoked within-subject variation in reported happiness also predicts the extent of probability weighting for the respective trials. Both the curvature and the elevation of the probability weighting function were modeled as depending on the condition-specific happiness ratings, whereas the curvature of the value function was assumed to be invariant across conditions. Our hypothesis was that both the between-subject and the within-subject effect would point in the same direction: the greater self-reported happiness, the lower the degree of probabilistic pessimism. The same procedure was used for the individual sadness and calmness ratings.

## Results

### Music-evoked incidental emotions

**Figure 2:**
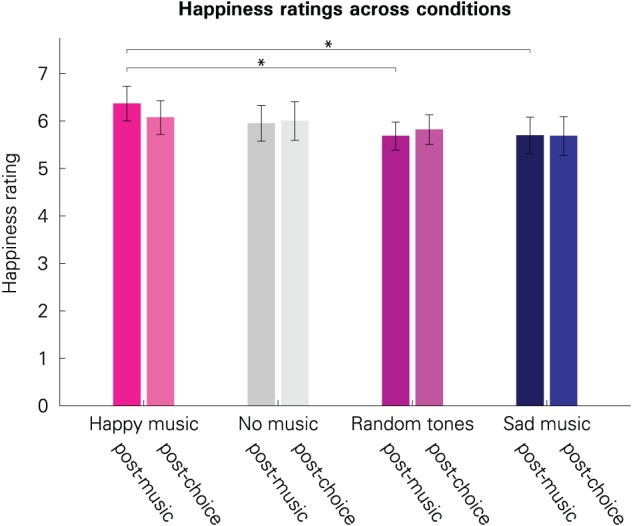
**Subjective happiness ratings across the four conditions.** Darker bars illustrate the values immediately after musical stimulation (“post-music”); brighter bars illustrate the values after the lottery choice blocks (“post-choice”). Error bars represent 95% confidence intervals. The scale ranged from 1 (not happy at all) to 9 (very happy). An asterisk indicates significant difference at the 5% level.

To test whether participants' emotional states were altered by our experimental manipulation, we compared the self-reported emotions between the conditions. As expected, participants' self-reported happiness was affected by the music that they had listened to (see Figure 2). Immediately after musical stimulation (“post-music”), participants' self-reported happiness varied significantly between conditions [*F*_(3, 120)_ = 2.745, *p* = 0.046]. This effect vanished until the second emotion rating at the end of a block, approximately 10 min later [“post-choice”; *F*_(3, 120)_ = 0.816, *p* = 0.487]. This is consistent with a diminishing intensity of evoked incidental emotions over time.

As expected, pairwise comparisons revealed that immediately after the musical stimulation, participants reported to be happier when they had listened to happy music than to sad music [*t*_(40)_ = 2.219, *p* = 0.032]. This also holds for the comparison between happy music and random tone sequences [*t*_(40)_ = 2.877, *p* = 0.006]. Reported happiness for random tone sequences was not significantly different from reported happiness for sad music [*t*_(40)_= −0.047, *p* = 0.962]. Taken together, this indicates that the “random tone sequences” condition was affectively more similar to the “sad” condition rather than being affectively neutral. No other differences were significant (all *p* > 0.149).

Mirroring the lowest happiness ratings, sadness ratings were highest in the “sad” condition. The within-subject effect for condition was only marginally significant, however, for the ratings taken directly after the musical stimulation [*F*_(3, 120)_ = 2.190, *p* = 0.093]. This trend toward significance might be due to a difference between the “sad” and “no music” condition [*t*_(40)_ = 2.41, *p* = 0.021], indicating that sad music was associated with greater sadness than no experimental manipulation (no music). The remaining comparisons were, however, not significant (all *p* > 0.152). Differences in the post-choice ratings were also not significant [*F*_(3, 120)_ = 1.759, *p* = 0.159].

Calmness ratings, which we consider an inverse indicator of arousal, did not show any significant post-music differences [*F*_(3, 120)_ = 1.435, *p* = 0.236] or post-choice differences [*F*_(3, 120)_ = 1.251, *p* = 0.294].

In summary, ratings reveal that music differentially altered the emotional state of happiness and that this effect diminished over time. Happy music was associated with greater happiness, whereas sad music and random tone sequences were associated with lower happiness compared to the “happy” condition.

### Lottery choices

#### Choice of the riskier lottery

**Figure 3:**
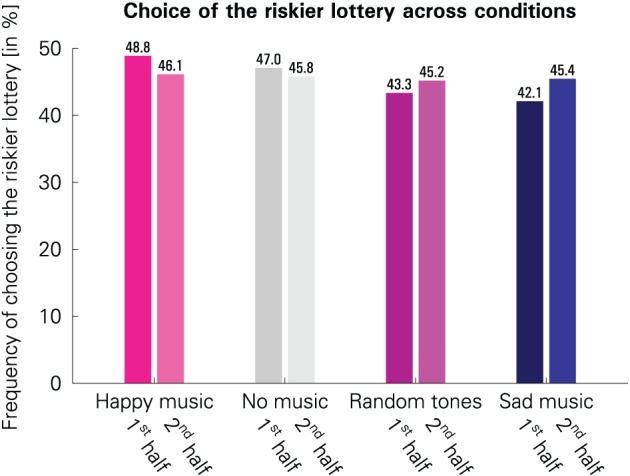
**Comparison of the frequencies with which the riskier lottery was chosen across the four conditions in the first 25 trials of a block following musical stimulation (1st half) and in the remaining 25 trials of a block (2nd half).** (Please note that this chart is shown only for illustrative purposes and is not used for statistical inference, because the statistical analysis needs to account for both between-subject and within-subject variation; see the description of LPM 1 and LPM 2 in the “Materials and methods” section.)

Participants chose the riskier lottery most often in the “happy” condition and least often in the “sad” condition. The relative frequencies of the riskier lottery being chosen in the four conditions are visualized in Figure 3.

Linear probability models (LPMs) were used to test whether these differences are statistically significant. In contrast to LPM 1, LPM 2 not only allows for analyzing the average effect of the conditions on choices, but it also permits analysis of the initial effects—i.e., the estimated frequency at which participants chose the riskier lottery immediately following musical stimulation—and time trends.

*F*-tests for overall condition effects were significant for both models [LPM 1: *F*_(3, 11444)_ = 4.7725, *p* = 0.0025; LPM 2: *F*_(3, 11440)_ = 4.8329, *p* = 0.0023], indicating differences in risk attitudes between the conditions. The results are presented in Table 1. As hypothesized, the “happy” and the “sad” condition were the two extreme conditions, with the riskier lottery being chosen most often in the “happy” condition.

**Table 1:** **Random-effects linear probability models for the choice of the riskier lottery across the four conditions**.

**Condition**	**LPM 1**		**LPM 2**	
	**Average frequency (%)**	**Average frequency (%)**	**Initial frequency (%)**	**Time trend (%)**
Happy music	47.40^tones^^,^^sad^	47.48^tones^^,^^sad^	50.50^tones^^,^^sad^	−0.12^0^^,^^tones^^,^^sad^
No music	46.48^tones^^,^^sad^	46.43^sad^	49.11^tones^^,^^sad^	−0.11^sad^
Random tones	44.20^happy^^,^^no^	44.20^happy^	43.12^happy^^,^^no^	+0.04^happy^
Sad music	43.72^happy^^,^^no^	43.75^happy^^,^^no^	40.27^happy^^,^^no^	+0.14^0^^,^^happy^^,^^no^

LPM 1 included only dummy regressors to detect differences between the conditions. In addition to that, LPM 2 also modeled the temporal distance from the last musical stimulation (as the number of trials completed since the last musical stimulation). The “time trends” column thus indicates by how much (in percentage points) the relative frequency at which the riskier lottery was chosen changed on average with each additional completed trial. t-tests were used to assess whether the parameter estimates are different from 0.

Significance at p < 0.05 indicated via superscripts:

**happy:** significantly different from the “happy music” condition;

no significantly different from the “no music” condition;

**tones:** significantly different from the “random tone sequences” condition;

sad significantly different from the “sad music” condition;

0 significantly different from zero (for the time trends).

To account for individual differences in participants’ risk taking, individual random effects were included for the respective reference condition.

These two conditions differed from each other significantly (*p* = 0.0013) in LPM 1. The “happy” condition also differed significantly from “random tones” (*p* = 0.0053). In addition, the “sad” (*p* = 0.0192) and the “random tones” condition (*p* = 0.0464) were associated with higher risk aversion than “no music.”

These results carry over to LPM 2, except that for the difference between “random tones” and “no music” there was only a trend toward significance (*p* = 0.0509). In LPM 2, the estimated initial effects—i.e., choice of the riskier lottery immediately after having listened to the music—are even more pronounced than the average effects in both LPMs.

The estimated time trends in LPM 2 show that participants became more risk-averse over time when they started out with relatively low risk aversion (i.e., in the “happy” and “no music” conditions), and they became less risk-averse over time when starting out with relatively high risk aversion (i.e., in the “random tone sequences” and “sad music” conditions). The time trends are significantly different from zero for the two most extreme conditions, i.e., the “happy” (*p* = 0.0256) and the “sad” condition (*p* = 0.0130), and there was a trend toward significance for no music (*p* = 0.0509). At the end of each block, there were no significant differences in the choice frequencies between conditions anymore (all pairwise *p* > 0.21). This is what one would expect for a diminishing emotional influence over time. An *F*-test rejects the hypothesis that time had no influence on choices [*F*_(4, 11440)_ = 3.8951, *p* = 0.0037]. The estimates for several conditions differed significantly from each other (see Table 1).

In summary, analysis of the relative frequency with which participants chose the riskier lottery provides evidence in favor of an influence of music-evoked incidental emotions on risk attitudes.

#### Structural regressions

To test our hypothesis that the influence of incidental emotions on risk attitudes can be explained through changes in probability weighting, we estimated preference parameters via structural regression models. First, we estimated a full model that simultaneously allowed for between-condition changes in the curvature of the value function (ρ) and in the probability weighting parameters (α, β). The full model revealed an overall (jointly) significant effect of music-evoked emotions on the estimated preference parameters [*F*_(9, 16304)_ = 3.1268, *p* = 0.0009].

Allowing for between-condition changes in the value function parameter (ρ) did, however, not significantly improve the model fit compared to a reduced model that only allowed for changes in the probability weighting parameters (log-likelihood ratio = 1.0159, *p* = 0.7974). This indicates that—as to be expected based on theoretical considerations—changes in the curvature of the value function do not explain additional variation in participants' decisions beyond what is explained by changes in probability weighting. As a consequence, we focused on the more parsimonious model^6^.

**Figure 4:**
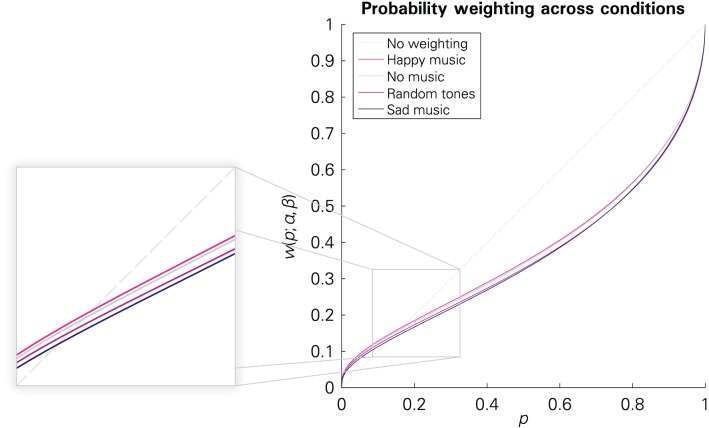
**Probability weighting functions in the four conditions based on the parameter estimates for the structural regression model reported in Table 2**.

According to this reduced model, there was a significant effect of music-evoked emotions on the estimated preference parameters [*F*_(6, 16307)_ = 4.5233, *p* = 0.0001]. Changes in the elevation parameter β were significant between the “happy” and the “sad” and the “happy” and the “random tones” condition, respectively (see Table 2), as well as between “sad” and “no music” (−0.0614; *p* = 0.001) and “random tones” to “no music” (−0.417, *p* = 0.0243)^7^. No between-condition changes in the sensitivity parameter α reached significance (all *p*-values > 0.49). That is, listening to happy music was associated with a significant increase in the elevation of the probability weighting function—i.e., higher (more optimistic) decision weights of the larger outcomes—compared to listening to random tone sequences and to sad music. Listening to sad music and random tones was also associated with lower (more pessimistic) decision weights than not listening to any music. The respective probability weighting functions are illustrated in Figure 4. A regression in which we interacted the between-condition regressors with a gender dummy revealed no significant difference in the effect of emotions on the probability weighting of men and women.

**Table 2:** **Structural regression model: estimates of preference parameters—sensitivity and elevation of the probability weighting function in the “happy music” condition as well as changes of the parameters in the remaining conditions**.

**Condition**	**Coefficient**	***p*−value**
ρ: **CURVATURE OF VALUE FUNCTION**		
Average over all conditions	0.2467	0.006
α: **SENSITIVITY OF PROBABILITY WEIGHTING FUNCTION**		
Happy music (reference condition)	0.5476	<0.001 (*H*_0_: α = 1)
Δ No music	+0.0035	0.864
Δ Random tones	−0.0105	0.603
Δ Sad music	+0.0017	0.934
**β : ELEVATION OF PROBABILITY WEIGHTING FUNCTION**		
Happy music (reference condition)	1.3003	0.002 (*H*_0_: β = 1)
Δ No music	+0.0154	0.392
Δ Random tones	+0.0576	0.002
Δ Sad music	+0.0769	<0.001
**σ : FECHNER NOISE**		
Average over all conditions	0.6945	<0.001

Wald tests were used to assess whether the parameter estimates are different from 0. While the benchmark for the curvature of the value function is 0 (ρ = *0* in the case of a linear value function), it is 1 for the other two parameters (α = *1* and β = *1* in the absence of probability weighting). Thus, except for α and β, each statistical test reported here was calculated under the null hypothesis (H_*0*_) that the coefficient equals 0. A decrease in α indicates a decrease in the sensitivity to variation in probability; an increase in β indicates a decrease in the elevation of the probability weighting function. Individual random effects were included in α, β, ρ, and σ, but not in the between-condition changes. A logit regression model was used. Please note that our results can be compared to studies that used *u*(x; r) = x^r^ by calculating *r* = *1* - ρ.

To assess the magnitude of the observed effects, it is useful to translate the changes in preference parameters into changes in monetary units. Based on the estimated preference parameters—including the individual random effects—and the estimated between-condition changes in these parameters, it is possible to calculate the (subjective) certainty equivalents of all the lotteries presented to the participants across trials. One can then calculate the risk premium for each lottery, which is defined as the difference between the expected value of a lottery and its certainty equivalent. When averaging across lotteries and across participants, we find that the mean risk premium implied by the estimated parameters is €1.34 (14.05% of the mean expected value) in the “sad” condition, while it is €1.24 (12.93%) in the “happy” condition. This means that the average risk premium is 8.17% (1.12 percentage points) higher after listening to the sad music compared to the happy music used in our experiment.

We further investigated the link between incidental emotions and risk attitudes in a complementary fashion by estimating participants' probability weighting parameters as functions of their individual happiness ratings (for the results, see Table 3). Put differently, the regression included two explanatory variables—between-subject differences in average happiness in the “no music” condition and within-subject deviation from this average resulting from the musical stimulation.

**Table 3:** **Structural regression model: estimates of preference parameters—sensitivity and elevation of the probability weighting function as functions of the between-subject and within-subject variation in self-reported happiness**.

**Self-reported happiness**	**Coefficient**	***p*−value**
**ρ : CURVATURE OF VALUE FUNCTION**		
Average over all conditions and participants	0.3418	<0.001
**α : SENSITIVITY OF PROBABILITY WEIGHTING FUNCTION**		
Average in “no music” condition over all participants	0.5900	<0.001 (*H*_0_: α = 1)
Deviation of participants’ average in “no music” condition from cross-subject mean (between-subject regressor)	+0.0093	0.683
Deviation of participants’ block-specific rating from “no music” condition (within-subject regressor)	−0.0221	0.180
**β : ELEVATION OF PROBABILITY WEIGHTING FUNCTION**		
Average in “no music” condition over all participants	1.1393	0.198 (*H*_0_: β = 1)
Deviation of participants’ average in “no music” condition from cross-subject mean (between-subject regressor)	−0.0437	0.143
Deviation of participants’ block-specific rating from “no music” condition (within-subject regressor)	−0.0853	0.003
**σ : FECHNER NOISE**		
Average over all conditions and participants	1.0471	<0.001

Wald tests were used to assess whether the parameter estimates are different from 0. While the benchmark for the curvature of the value function is 0 (ρ = *0* in the case of a linear value function), it is 1 for the other two parameters (α = *1* and β = *1* in the absence of probability weighting). Thus, except for α and β, each statistical test reported here was calculated under the null hypothesis (H_*0*_) that the coefficient equals 0. A decrease in α indicates a decrease in the sensitivity to variation in probability; an increase in β indicates a decrease in the elevation of the probability weighting function. The standard errors—and thus the associated p-values—were adjusted for 41 clusters on the subject level. A logit regression model was used. Please note that our results can be compared to studies that used u(x; r) = x^r^ by calculating *r* = *1* - ρ.

We found a significant relationship between the within-subject regressor—i.e., the music-evoked variation in happiness—and the elevation of the probability weighting function (*p* = 0.003). Specifically, the happier participants were, the more elevated their probability weighting function was, resulting in decreased risk aversion with increasing happiness.

As expected, this pattern was also found for the between-subject variation observed in the “no music” condition (although here the associated coefficient did not reach significance, *p* = 0.143): participants who were happier in the “no music” condition tended to be less risk-averse, indicated by a more elevated probability weighting function.

While the latter—between-subject—finding is correlational, the former—within-subject—finding again supports the interpretation that evoked emotions *causally* influence risk attitudes.

As far as calmness is concerned, we only found a trend toward significance for participants who were overall less calm/more aroused to have a more elevated probability weighting function (*p* = 0.068). Importantly, we did not observe an analogous effect for the music-evoked (within-subject) changes in arousal (*p* = 0.276). We also did not find any significant effect of self-reported sadness on the elevation parameter of the probability weighting function—neither for the between-subject regressor (*p* = 0.666) nor for the within-subject regressor (*p* = 0.185). Hence, we found happiness to be the only emotional experience that was related to the elevation parameter of the probability weighting function at the individual level.

In summary, the results of our structural regressions confirmed the observed differences in how often the riskier lottery was chosen in the “happy” condition on the one hand and the “sad” and “random tone sequences” conditions on the other hand. Importantly, however, the structural regressions go beyond that by showing that the changes in participants' choices can be explained through changes in how they convert objective probabilities into subjective decision weights—in particular through changes in the elevation parameter of the assumed probability weighting function. The hypothesized affective nature of this link is corroborated by our finding that both self-reported happiness in the “no music” condition and music-evoked changes in happiness were positively related to the elevation of the probability weighting function and thus negatively related to risk aversion.

## Discussion

Cumulative prospect theory (CPT; Tversky and Kahneman, 1992) is a theory of decision making under risk that is very prominent in both psychology and economics. In this framework, risk attitudes are understood as arising from an interplay between subjective valuation of (monetary) outcomes and probability weighting. Previous studies have demonstrated an affect-congruent influence of incidental emotions on the assessment of unknown probabilities of potential events, for example, more optimistic judgments in happy participants and more pessimistic probabilistic judgments in sad participants (Johnson and Tversky, 1983; Wright and Bower, 1992). We hypothesized that such an effect would also exist on probability weighting in decision making under risk.

We found experimental evidence in favor of a *causal* effect of incidental emotions on risk attitudes that is consistent with changes in probability weighting. To measure risk attitudes and probability weighting, we employed a variant of the Random Lottery Pairs procedure (Hey and Orme, 1994) and varied both outcomes and probabilities of real monetary gambles in the gain domain. Participants' incidental emotions were manipulated within-subject by listening to happy and sad music as well as random tone sequences or no music at all, and evaluated by self-reported emotional ratings.

Our two-step statistical analysis yielded that participants' decisions differed between conditions and that these differences can be explained by changes in probability weighting. First, we compared the choice frequencies between conditions. Risk aversion decreased from the “happy” to the “random tones” and “sad” conditions. Second, we allowed for emotion-dependent changes in the probability weighting function in a structural regression rooted in CPT. We found a significantly higher elevation in the “happy” than in the “sad” and the “random tones” condition. That is, participants made decisions as if the probabilities of the larger payoffs received a higher decision weight in the “happy” condition and lower weights in the other two. This could be regarded as a form of optimism or pessimism, respectively. Listening to sad music and random tones was also associated with more pessimism than not listening to any music. The sensitivity parameter was not affected. Thus, affectively mediated changes in risky choices do not seem to result from altered sensitivity to probability changes but from a change in decision weights across probabilities.

Several arguments support the claim that these effects can be attributed to incidental emotions. First, the effects correspond closely to differences in self-reported happiness between these conditions. Happy music was associated with greater happiness, whereas sad music and random tones were associated with decreased happiness. Second, we found that the effect on decisions diminished over time—just as the effect on self-reported happiness. Third, music-evoked happiness correlated positively with the estimated elevation of the probability weighting function: when happiness was greater, the larger payoffs received a higher decision weight; when happiness was reduced, the larger payoffs received a lower decision weight. Taken together, this evidence is compatible with an effect of incidental emotions on the elevation of the probability weighting function during decision making under risk.

Our results are consistent with well-established effects of incidental emotions on probability judgments reported in the psychological literature. For instance, happy people make more optimistic probabilistic judgments, while sad people make more pessimistic judgments (Johnson and Tversky, 1983; Wright and Bower, 1992). Extending this body of evidence, our results suggest that not only judgments of unknown probabilities are altered, but that also the weighting of known probabilities in decision making under risk is affected by incidental emotions.

This is in line with indirect evidence that suggests an effect of incidental emotions on probability weighting. In a correlational study, Fehr-Duda et al. (2011) found that women that regarded the current day to be more promising than usual made decisions as if they weighted the larger payoffs more optimistically. This has been interpreted as an effect of mood on the elevation of the probability weighting function in women. In a similar vein, weather and seasonal effects on decision making were attributed to the effect of bad mood on probability weighting (Kliger and Levy, 2008)—importantly, however, without distinguishing between sensitivity and elevation of the probability weighting function.

We complement this research in important ways by going beyond correlational data and providing evidence in favor of a causal effect of incidental emotions on risk attitudes that is consistent with changes in probability weighting in particular. Critically, we experimentally manipulated participants' incidental emotions. Moreover, we recorded participants' self-reported emotions to make sure that the experimental manipulation worked as intended. Taken together, this is evidence in favor of a causal effect of incidental emotions. Unlike Fehr-Duda et al. (2011), we found significant effects for our whole mixed-gender sample and no significant difference between men and women. Thus, gender does not seem to be the major determining factor in the effect of emotions on risk attitudes. Similar to the correlational evidence reported in Fehr-Duda et al. (2011), increased baseline happiness was associated with a more elevated probability weighting function, although not significantly so. This between-subject effect is also in line with the finding that people with high life satisfaction are more willing to take risks (German Socio-Economic Panel; Dohmen et al., 2011).

At first glance, our results may seem inconsistent with the findings of Isen et al. (1988). Isen et al. did not report a significant effect of evoked positive affect on risk attitudes in the gain domain. Their Figure 1 displays estimated utility functions whose curvature is less pronounced in the gain domain for the positive-affect than for the control participants. This would be consistent with reduced risk aversion over gains resulting from positive affect. However, no statistical test was performed to determine whether this difference was significant. Nevertheless, Isen et al. speculated that in the gain domain there might be a tendency for reduced risk aversion based on more optimistic probability weighting in happy participants. Importantly, our study provides empirical evidence for this very conjecture.

Previous research has already provided some theoretical accounts on the affect sensitivity of the probability weighting function. The inverse S-shape of the probability weighting function can result from the presence and integration of anticipatory emotions—e.g., elation and disappointment—in the decision process (Gul, 1991; Brandstätter et al., 2002; Walther, 2003). For instance, Brandstätter et al. (2002) demonstrated that an inverse S-shaped probability weighting function can be reconstructed from a so-called surprise function that reflects participants' measured anticipated happiness with regard to the outcome. In this framework, the anticipated disappointment that might result from a failure to achieve a highly probable gain is thought to translate into lower decision weights for high probabilities. In line with this, probability weighting was found to be more pronounced for outcomes believed to elicit stronger emotional responses (Rottenstreich and Hsee, 2001). However, it has been pointed out that anticipatory emotions could theoretically also alter the elevation of the function at each probability (Rottenstreich and Hsee, 2001). Our results indicate that not just anticipatory, but also incidental emotions contribute to probability weighting and that this is reflected in the elevation of the function. Incidental emotions might have a direct effect on the processing of probabilities, leading to optimism/pessimism in terms of decision weights. Alternatively, they might (also) operate through changing anticipatory emotions that affect the elevation of the probability weighting function indirectly. Given that we used very moderate and only positive monetary outcomes that are unlikely to create strong positive or negative anticipatory emotions—compared to stimuli used in other experiments, like receiving a kiss or a painful electric shock (Rottenstreich and Hsee, 2001)—we favor the former interpretation, but we cannot rule out the latter, indirect, channel.

Our research has several implications for future research. We have demonstrated that incidental emotions influence choices between monetary gambles in a way that is compatible with emotion-induced changes in the subjective weighting of known probabilities. An important next step would be to explore the underlying mechanism in greater detail. Process-tracing methods like eye tracking or (computer) mouse tracking might offer deeper insights into the psychological processes that underlie decision making (e.g., Schulte-Mecklenbeck et al., 2011) and affective influences. For instance, it has been shown that happy participants have a stronger attentional focus on rewards (Tamir and Robinson, 2007). It is possible that probability weighting ultimately reflects changes in attention to outcome values, as has also been pointed out by Wu (1999).

Neural data are another promising source of information. Different brain areas have been related to the processing of the basic components of gambles, i.e., of reward magnitude and probabilities (Tobler et al., 2007). Concerning probability weighting, previous research has associated non-linear probability weighting with non-linear neural responses in the striatum and anterior cingulate cortex (Paulus and Frank, 2006; Tobler et al., 2008; Hsu et al., 2009). Hence, if it is indeed probability weighting that is affected by incidental emotions, we should see emotion dependence of these neural responses. For instance, the striatum and anterior cingulate cortex are also associated with experiencing happiness, as a meta-analysis of studies on emotional processing revealed (Vytal and Hamann, 2010). A link between rewards and emotions is also plausible, given the association between activity in the striatum and anticipation of rewards as well as self-reported happiness generated by these rewards (Knutson et al., 2001). It is also possible that emotions not related to the decision at hand—i.e., incidental emotions—have an influence on reward processing in the striatum. It has been suggested that conditioned and unconditioned stimuli—and this would include a wide range of emotional stimuli evoking incidental emotions—influence instrumental, reward-based behavior via the ventral striatum (Cardinal et al., 2002). Recently, pleasurable music has been shown to facilitate reward-based learning, and the observed effect seems to be linked to striatal activation (Gold et al., 2013). Thus, we would expect that incidental emotions influence decision making, and probability weighting in particular, by altering activity in these brain areas that show such a functional overlap in reward and emotion processing. This might reflect the direct integration of incidental emotions into the decision process.

Obtaining neurobiological measures of emotion-induced changes in probability weighting are highly promising for future research, given that from the observation of choices alone, it is impossible to disentangle changes in the value function from changes in probability weighting when using two-outcome lotteries only (Wakker, 2010, chapter 5)—unless one restricts the involved functions to specific parametric forms (in our case, power utility in combination with Prelec-style two-parameter probability weighting). Specifically, reduced elevation of the probability weighting function is observationally equivalent to an increase in the curvature of the value function: both lead to increased risk aversion. As a consequence, our findings (and those of Fehr-Duda et al., 2011) are *consistent* with changes in probability weighting and thus our hypotheses, but *not exclusively so*, since the between-condition differences in our participants' choices can also be captured by the value function. The analysis of process or neural data might help disentangling the two possible effects.

Apart from those implications for future research, there are other methodological considerations and potential limitations more directly related to our research approach. In contrast to several previous studies on the influence of incidental affect on decision making that used between-subject designs (see, however, e.g., Knutson et al., 2008; Guitart-Masip et al., 2010), we employed a within-subject design because it has several advantageous features. First, our within-subject design is an ecologically more valid abstraction of the everyday decision environment of a person that is confronted with the same decision or similar decisions repeatedly while being in different affective states. In contrast, a between-subject design looks at different persons who make fewer decisions in only one affective state each. Second, a within-subject design increases statistical power because it prevents between-condition variance from being contaminated by between-subject variance.

Although within-subject designs potentially introduce confounds (e.g., via learning/time trends across sessions), there are reasons to believe that internal validity with respect to the effects of interest to our study is ensured, given that learning would rather diminish than exacerbate between-condition differences^8^. Taken together, we think that the experimental design that we used creates a balance between ecological and internal validity.

Regarding the set of lottery pairs, we focused, as already mentioned, on the gain domain for the following reasons: first, neuroimaging and lesion studies suggest that losses and gains are processed differently in the human brain (De Martino et al., 2010; but see Tom et al., 2007). Second, to increase the power for the detection of an effect, a sufficient number of decision trials is needed. Third, mixed gambles would have required the estimation of additional parameters, making even more observations necessary. We therefore deliberately chose to dedicate all our experimental trials to only one domain. However, the neuroimaging results just mentioned as well as evidence that probability weighting might be different in losses (Abdellaoui, 2000) should motivate future research to investigate the effects of incidental emotions on decision making in the loss domain.

A final remark on our emotional manipulation procedure is in order. While the music we used was able to evoke different levels of happiness, at a small to medium-sized effect, sadness was not reliably altered (which means that the sad music that we used was associated with decreased levels of happiness rather than greater sadness). Other emotion induction techniques might be more potent and also promising for future research (Gross and Levenson, 1995; Rottenberg et al., 2007). Alternatively, letting participants bring their own personal music that they know to evoke the desired emotional state might be a more potent form of induction, although the use of non-standardized, highly variable stimuli and inadvertently providing information about the study design to participants in advance might introduce various confounds.

Apart from this, different measures of emotional change—for instance, visually supported assessment scales like the Self-Assessment Manikin (Bradley and Lang, 1994) or psycho-physiological measures (e.g., skin conductance response or facial electromyography)—could be used, because participants might find it difficult to report their affective states on a numbered scale. In addition, one could focus on the underlying appraisal dimensions of emotions (see, e.g., Lerner and Keltner, 2000, 2001). In this regard, we have found preliminary evidence that arousal is not the causal emotional dimension, since we did not find a significant within-subject association between the calmness ratings (our inverse proxy for arousal) and risk attitudes.

The type and strength of emotional manipulation in our study is especially interesting given that everyday life is characterized by the exposition to many emotional stimuli that are not extreme in most cases (e.g., listening to music, being smiled at, or meeting more or less liked colleagues; compared to, say, winning a world championship, witnessing a terrorist attack, or losing a loved one). Hence, our design has ecological validity with respect to decision making occurring under standard affective contexts, i.e., small to moderate emotional changes.

We consider this just as interesting as investigating the effects of rather big, but uncommon, emotional changes. Intense feelings, especially when being fully recognized, can result in reduced emotional effects on decision making via an enhanced ability to control emotional bias (Seo and Barrett, 2007). Even more intense changes in emotion might result in avoiding making a decision altogether and postponing it to less turbulent times. In contrast, people may be relatively unaware of the influence of subtle emotional changes on their decisions and hence may be unable to regulate it. We therefore consider investigating the consequences of subtle, but common changes in incidental emotions highly relevant.

## Conclusion

Our study investigated within-subject the effects of incidental emotions on probability weighting by means of experimental manipulation and through measurement of changes in the affective state. We thereby complement previous studies on the effect of incidental emotions on probability judgments as well as previous—correlational—studies on the link between emotional states and probability weighting in decision making under risk. We found experimental evidence in favor of a causal influence of incidental happiness on risk attitudes. Via structural regressions based on CPT, we showed that these changes in risk attitudes can be attributed to affectively mediated changes in the elevation of the probability weighting function.

### Conflict of interest statement

The authors declare that the research was conducted in the absence of any commercial or financial relationships that could be construed as a potential conflict of interest.

## Footnotes

^1^We obtain qualitatively identical results if we consider variance as the risk measure—i.e., if we take the presence of a mean–variance trade-off as the criterion for one lottery (the one with the higher variance, but also the higher average payoff) to be riskier than the other. A wider spread implies increased variance, but not vice versa, so that the two measures coincide in many but not all of our trials.

^2^Using a probit or logit model instead of the linear probability model yields qualitatively identical results. However, the estimates obtained via an LPM are easier to interpret.

^3^We tried different specifications of the utility function. All yielded comparable fits, but power utility performed best (in line with the findings by Stott, 2006).

^4^To be precise, we used power utility of the form *u*(*x*; ρ) = (*x*^1 − ρ^− 1)/(1 − ρ). This is completely equivalent to the often used *u*(*x; r*) =*x*^*r*^, with *r* = 1 − ρ. The main advantage of using 1 −ρ as the exponent is the intuitive meaning of ρ: an increase in ρ indicates an increase in risk aversion—whereas for *x*^*r*^, a decrease in *r* indicates an increase in risk aversion.

^5^Using a probit instead of a logit specification leads to negligible changes. Like Stott (2006), we found the logit model to provide the best fit.

^6^We did not include time trends in this model, because this would have doubled the already large number of probability-weighting–related parameters to be estimated from 8 to 16.

^7^The between-condition changes in the elevation parameter β remain significant also when not allowing α to vary between the conditions.

^8^When checking for time trends across experimental sessions, we found that subjects became significantly more consistent over time (i.e., the Fechner error, σ, exhibited a significantly negative time trend), but the estimated probability weighting parameters showed no significant time trends. Moreover, the results did not differ qualitatively from those reported in Table 2. To address potential order effects, we checked whether the initial condition had a lasting influence on risk attitudes—which was not the case: when averaging the individual random effects depending on the condition that subjects first participated in, the resulting order of the average random effects in the elevation parameter for the four conditions corresponded in no way to the order of the respective estimated condition differences.

## Appendix

**Table A1:** **Set of lottery pairs**.

**No**.	**Lottery *A***			**Lottery *B***		
	***x_A,1_***	***x_A,2_***	***p_A,1_***	***x_B,1_***	***x_B,2_***	***p_B,1_***
1	5	10	0.25	6	9	0.10
2	5	10	0.25	6	9	0.25
3	3	15	0.50	6	9	0.25
4	4	15	0.50	6	9	0.50
5	3	15	0.50	5	10	0.90
6	3	15	0.50	6	10	0.75
7	2	20	0.50	4	12	0.75
8	3	15	0.50	7	9	0.50
9	4	15	0.50	7	9	0.75
10	2	20	0.75	5	10	0.90
11	2	20	0.75	3	15	0.25
12	2	20	0.75	4	15	0.75
13	7	12	0.75	8	10	0.50
14	3	15	0.50	7	12	0.50
15	3	15	0.50	6	12	1.00
16	6	15	0.75	7	9	0.50
17	7	15	0.75	7	9	0.10
18	6	15	0.75	8	8	0.50
19	6	15	0.75	9	9	0.50
20	3	15	0.50	8	10	0.25
21	3	14	0.50	8	10	0.10
22	3	15	0.50	6	9	0.50
23	3	15	0.75	5	10	0.10
24	2	20	0.50	5	10	0.50
25	3	20	0.50	5	10	0.25
26	6	15	0.75	6	10	0.50
27	6	14	0.75	6	10	0.50
28	3	15	0.50	8	8	0.10
29	3	15	0.50	7	7	0.25
30	3	15	0.50	6	15	0.25
31	3	14	0.50	6	15	0.10
32	6	12	0.50	7	9	0.50
33	6	12	0.50	7	9	0.25
34	8	15	0.75	6	10	0.10
35	8	15	0.90	6	10	0.90
36	3	15	0.50	7	9	0.50
37	4	12	0.50	7	9	0.10
38	3	15	0.25	11	11	0.25
39	3	15	0.10	11	11	0.50
40	6	9	0.25	8	10	0.50
41	6	9	0.25	8	10	0.90
42	3	15	0.25	4	12	0.90
43	3	15	0.25	5	12	0.10
44	4	12	0.50	6	9	0.50
45	5	12	0.50	6	9	0.25
46	4	12	0.10	11	11	0.50
47	4	12	0.25	11	11	0.75
48	4	12	0.50	7	12	0.50
49	4	12	0.25	6	10	0.90
50	4	12	0.25	6	11	0.10
51	3	15	0.50	6	9	0.25
52	4	15	0.50	6	9	0.25
53	3	15	0.50	7	9	0.50
54	6	12	0.50	6	9	0.90
55	6	12	0.50	6	9	0.75
56	6	12	0.75	6	9	0.75
57	2	20	0.25	13	13	0.50
58	4	20	0.25	13	13	0.75
59	3	15	0.50	7	12	0.90
60	3	15	0.50	7	13	0.25
61	4	12	0.25	8	15	0.75
62	4	12	0.25	8	16	0.50
63	2	20	0.10	17	17	0.50
64	2	20	0.10	16	16	1.00
65	2	20	0.50	7	9	0.50
66	2	20	0.50	7	9	0.10
67	4	12	0.25	9	13	0.50
68	4	12	0.25	9	13	0.50
69	4	12	0.25	5	10	0.25
70	4	12	0.25	5	10	0.10
71	2	20	0.50	7	12	0.50
72	2	19	0.50	6	9	0.10
73	2	20	0.50	6	9	0.50
74	3	15	0.10	13	13	0.25
75	3	15	0.10	13	13	0.50
76	5	10	0.25	7	9	0.50
77	5	10	0.25	7	9	0.10
78	2	20	0.50	3	15	0.25
79	2	20	0.50	3	14	0.25
80	3	15	0.50	4	12	0.10
81	3	15	0.50	5	12	0.50
82	2	20	0.50	4	12	0.25
83	2	20	0.50	4	11	0.10
84	3	15	0.75	4	15	0.90
85	3	15	0.75	5	15	0.50
86	5	10	0.10	9	13	0.10
87	5	10	0.10	9	13	0.25
88	6	10	0.10	9	13	0.50
89	6	11	0.25	4	7	0.50
90	6	12	0.75	4	7	0.90
91	6	11	0.50	3	8	0.90
92	6	11	0.75	3	8	0.10
93	6	8	0.50	3	5	0.50
94	6	9	0.75	4	9	0.25
95	5	9	0.50	4	8	0.50
96	5	5	1.00	3	5	0.75
97	5	7	0.50	4	6	0.50
98	4	6	0.25	3	5	0.90
99	5	8	0.10	5	8	0.75
100	5	7	0.50	4	6	0.75

**Table A2:** **Musical stimuli**.

**Category**	**Composer (Artist)**	**Title**
Happy	Joel Francisco Perri	El Canto de Mi Antara
	Craobh Rua	The Lucky Penny
	Scotch Mist	Shetland Tune
	Alfredo de Angelis	Pregonera
	Romanian Folk Dance	Batuta de la Adancata
	Louis Armstrong	St. Louis Blues
	Niccoló Paganini	Violin Concerto No. 1, 3rd movement
	Jonathan Richman	Egyptian Reggae
	Johann Joachim Quantz	Concerto for Flute and Orchestra, No. 256 in A Major – allegro Di Molto
	Franz Anton Hoffmeister	Concerto for viola and orchestra in D major: I. Allegro
	Georg Friedrich Händel	Arrival of the Queen of Sheba (Sinfonia from the opera Solomon)
	Michael Praetorius	Dances from Terpsichore: 6. Volte
Sad	Samuel Barber	Adagio for Strings
	Goran Bregovic and Athens Symphony Orchestra	Elo Hi (Canto Nero)
	Himlar Örn Hilmarsson	The Black Dog and the Scottish Play
	Frédéric Chopin (1837)—Alfred Eschwé and Razumovsky Sinfonia	Marche funebre from Piano Sonata No. 2 in B Flat Minor, Op. 35
	The Cure	Trust
	The Cure	Apart

**Table A3:** **Statements used in the emotion ratings**.

**German original**	**English translation**
Ich bin ruhig.	I am calm.
Ich bin sehr neugierig.	I am very curious.
Ich habe alles unter Kontrolle.	I have everything under control.
Ich bin fröhlich.	I am happy.
Ich bin traurig.	I am sad.
Ich führe ein stressiges Leben.	I lead a stressful life.
Ich fühle mich wohl.	I am comfortable.
Ich bin entspannt.	I am relaxed.
Ich fühle mich sicher.	I feel safe.
Letzte Nacht habe ich gut geschlafen.	I slept well last night.

### Theoretical note

We pointed out that for two-outcome lotteries, it is impossible in the framework of CPT to dissociate the shape of the value (utility) function from the shape of the probability weighting function (see Wakker, 2010, chapter 5), unless one restricts the functions to specific parametric forms.

To be precise, Wakker (2010) shows that the observational equivalence—or “data equivalence,” as he calls it—between expected utility with a non-linear utility function and rank-dependent utility (CPT in the gain domain) with a non-linear probability weighting function holds for two-outcome lotteries for which the lower outcomes are zero. This can be easily extended, however, as follows:

Let *e* be the certainty equivalent of a two-outcome lottery that pays a high payoff *x*_*h*_ with probability *p*_*h*_ and a low payoff *x*_l_, where *x*_*h*_ > *x*_*l*_ ≥ 0, with probability 1 − *p*_*h*_. Then, for expected utility, we have *u*(*e*) = *p*_*h*_ *u*(*x*_*h*_) + (1 − *p*_*h*_) *u*(*x*_*l*_). Normalizing *u*(*x*_*h*_) = 1 and *u*(*x*_*l*_) = 0, this reduces to *u*(*e*) =*p*_*h*_. Under the assumption that *u*(*x*) is strictly increasing in *x*, this can be rewritten as *e* = *u*^−1^(*p*_*h*_), where *u*^−1^ denotes the inverse function of *u*.

Now consider the same certainty equivalent *e* to be instead generated by cumulative probability weighting with weighting function *w*(*p*) in combination with linear utility. This generates the equality *e* = *w*(*p*_*h*_) *x*_*h*_ + [1 − *w*(*p*_*h*_)] *x*_*l*_. Rearranging yields *w*(*p*_*h*_) = [*e* − *x*_*l*_]/[*x*_*h*_ − *x*_*l*_] = [*u*^−1^(*p*_*h*_) − *x*_*l*_]/[*x*_*h*_ − *x*_*l*_]. Since *u*^−1^(0) = *x*_*l*_ and *u*^−1^(1) = *x*_*h*_, the *w*(*p*_*h*_) found in this way ranges from 0 to 1 and is thus a perfectly valid probability weighting function. It takes on the shape of the inverse function of the utility function *u*(*x*), normalized in a suitable way.

Thus, the observational equivalence between expected utility with a non-linear utility function and rank-dependent utility with a non-linear probability weighting function holds for arbitrary two-outcome lotteries—and not only for those whose lower outcome is zero.

Disentangling the shape of the value (utility) function and the probability weighting function based on observed choices alone becomes possible when using lotteries that consist of at least three outcomes. However, doing so will still be hard statistically, since the parameters to be estimated continue to be interdependent, albeit to a lesser degree. For this reason, we suggest (see Discussion) to obtain additional non-choice data such as process-tracing data or neural data that might help to disentangle the underlying processes.
